# Associations between work experiences, health, well-being, and intention to remain in the profession among healthcare and social work professionals – a multicentre cross-sectional study

**DOI:** 10.1186/s12913-026-15575-y

**Published:** 2026-09-17

**Authors:** Isabelle Andersson Hammar, Sandra Pennbrant, Håkan Nunstedt, Inger Ahlstrand, Aimée Ekman, Jenny Hallgren, Ingrid Larsson, Margaretha Larsson, Qarin Lood, Annelie J. Sundler, Anna M. Johnsen

**Affiliations:** 1 Department of Health and Rehabilitation, Institute of Neuroscience and Physiology, The Sahlgrenska Academy, University of Gothenburg, Arvid Wallgrens backe, House 2, Box 455, Gothenburg, 405 30 Sweden; 2 Department of Health Sciences, University West, Trollhättan, Sweden; 3 School of Health and Welfare, Jönköping University, Jönköping, Sweden; 4 School of Health Sciences, University of Skövde, Skövde, Sweden; 5 Department of Health and Care, School of Health and Welfare, Halmstad University, Halmstad, Sweden; 6 Faculty of Caring Science, Work Life and Social Welfare, University of Borås, Borås, Sweden; 7 Swedish Red Cross University, Stockholm, Sweden

**Keywords:** Health and well-being, Intention to remain in the profession, Occupational health, Sustainable working life, Work experience

## Abstract

**Background:**

The relationship between work experiences, health, well-being, and job satisfaction is important, especially in high-stress professions like healthcare and social work. While work stress effects are well studied, less is known about how work experiences in early professional life impacts health, well-being, and intention to remain in the profession. Therefore, this study aimed to explore associations between work experiences, health, well-being, and the intention to remain in the profession among healthcare and social work professionals, three years after graduation.

**Methods:**

The study had a multicentre, cross-sectional design. As part of a larger longitudinal project on health-promoting factors during education and early careers, 165 of 1,751 invited graduates (from biomedical science, dental hygiene, nursing, occupational therapy, physiotherapy, radiology nursing, and social work) completed an online survey between December 2024 and January 2025. Instruments included the Work Experience Measurement Scale (WEMS), Sense of Coherence (SOC-13), and the Salutogenic Health Indicator Scale (SHIS), alongside questions on general health and well-being, physical activity, daily activity, sleep quality, and intention to remain in the profession. Data were analysed using descriptive statistics, non-parametric tests, and regression analyses.

**Results:**

Higher WEMS total scores were associated with better general health, better well-being, and a higher intention to remain in the profession. Supportive work conditions and internal work experiences were most strongly associated with intention to remain in the profession. Autonomy enhanced health and well-being but did not directly affect intention to remain in the profession. Participants aged 30-36 years reported a lower intention to remain in the profession than those in the younger and older age groups.

**Conclusions:**

Positive work experiences may be important for supporting health, well-being, and intention to remain in the profession among healthcare and social work professionals three years after graduation. Lower intention to remain in the profession among those aged 30-36 years suggests a potential need for targeted support during this specific stage of working life. The findings support the integration of early-career support, sustainable leadership, and supportive work conditions into workforce strategies across healthcare professions and social work.

**Supplementary Information:**

The online version contains supplementary material available at https://doi.org/10.1186/s12913-026-15575-y.

## Introduction

The relationship between work experience, health, well-being, and job satisfaction is a critical area of research, particularly in high-stress professions such as healthcare and social work [1]. Well-being at work is the overall condition of employees’ physical, mental, and social health shaped by the workplace covering safe working conditions and opportunities to build health and social well-being [2]. World Health Organisation (WHO) similarly emphasizes “healthier, safer and more resilient workplaces”, enabling people to do their jobs without work-related illness or injury, enhancing physical and mental health and social well-being, protecting harmony with nature, and providing protection in community disasters [3, 4]. Job satisfaction, on the other hand, is defined as the degree to which people feel content and fulfilled with their work, reflecting positive emotional responses to their job roles and work environment [5]. Job satisfaction is the positive emotional and attitudinal state that results from people’s appraisal of their job experiences. It reflects fulfilment, motivation, and overall happiness, shaped by factors such as the nature of the work, perceived fairness, recognition, growth opportunities, and quality interpersonal relationships. Job satisfaction is closely connected to commitment and a sense of belonging at work [6]. To improve well-being and job satisfaction, organisations should focus on safe and healthy work environments, decent work conditions, supportive colleague relationships, and opportunities for personal growth and achievement [7]. Healthcare and social work professions are characterised by demanding workloads, emotional strain, and continuous interaction with people who are facing complex challenges [8]. While the negative effects of work-related stress on physical and mental health are well established [9], less is known about how work experiences in early professional life are associated with health, well-being, and intention to remain in profession. Understanding these relationships is essential for improving workplace conditions and supporting long-term sustainability in these sectors. International organisations such as the World Health Organization (WHO), International Labour Organization (ILO), National Institute for Occupational Safety and Health (NIOSH), and the European Agency for Safety and Health at Work (EU-OSHA) emphasise the importance of healthy and sustainable working environments. These organisations highlight psychosocial working conditions, employee well-being, job satisfaction, leadership, and workforce retention as key factors influencing occupational health and the sustainability of health and social care services. In Sweden, sustainable working conditions and workforce retention are highlighted by agencies such as the Swedish Work Environment Authority [10] and the Public Health Agency of Sweden [11].

A sustainable work-life is one that remains viable in both the short and the long term for people, organisations, and society. For healthcare and social work, this requires conditions that support long-term health, motivation, and effective performance without adverse consequences [12, 13]. However, such conditions are often undermined by high workloads, limited recovery opportunities, emotional strain, administrative burdens [14], and conflicts between professional ethics and organisational goals [15].

Research highlights the importance of factors such as effective leadership, influence over decision-making processes [16], social support from colleagues, and opportunities for supervision and competence development [17]. Job satisfaction is also central as it has been shown to be closely linked to both intentions to remain in the profession and perceived quality of working life [18], while high staff turnover negatively affects quality of care, patient safety, and the work environment [19]. Promoting people’s intention to remain in the profession is therefore a key component for a sustainable working life in healthcare and social work.

Antonovsky’s [20, 21] salutogenic framework provides a useful lens for understanding how people manage stress. It introduces the concept of sense of coherence (SOC), defined as a person’s enduring confidence in the predictability of life and the availability of resources to meet its demands. Research shows that people with a strong SOC are better equipped to cope with stress [22, 23]. In work-life, resources such as balanced demands, participation in decision-making, and supportive relationships can therefore contribute to sustained health and well-being. Health and well-being are shaped by both individual and contextual factors, including lifestyle, mental health, social support, and working conditions [24–26].

In healthcare and social work, organisational demands and emotional strain place particular pressure on professionals [14, 15]. Well-being is further linked to the fulfilment of basic psychological needs for autonomy, competence, and relatedness [27]. Autonomy is a central dimension of the psychosocial work environment and is commonly understood as employees’ perceived influence over their work tasks, methods, and decision-making in everyday practice [28].

A substantial body of research highlights the importance of supportive work environments for professionals’ intention to remain in the profession. For instance, support from experienced colleagues has been shown to strengthen early-career professionals’ confidence and competence, increasing their intention to remain in the workforce [16, 17, 29–31], as well as manageable demands identified as key predictors of intention to remain in the profession [32]. Organisational factors such as flexibility, opportunities for autonomy [16], professional development [33], further contribute to professionals’ intention to remain in the profession. Practices including social support from colleagues and supervisors, as well as high-quality leadership, have been linked to nurses’ well-being and reduced psychological distress, factors that may support continued participation in the profession [34]. Supportive working conditions are also central to maintaining workplace health. Adequate resources, safe environments, and positive collegial relationships enhance employees’ sense of support [35]. Work-related resources, such as positive work experiences, work-life balance, opportunities for recovery [36], and supportive interactions with colleagues, supervisors, and patients [37], are essential for sustaining a healthy work environment [35]. In high-stress professions, the interplay between these resources and employees’ experiences of working life is particularly important for both well-being and job satisfaction [16].

While previous literature has focused on negative effects of work-related stress [9, 38], less attention has been given to how work experiences during early professional life relate to health, well-being, and professionals’ intention to remain in the profession [38–40]. To address this gap, the present study aimed to explore associations between work experiences, health, well-being, and intention to remain in the profession among healthcare and social work professionals three years after graduation. The following research questions were formulated:

1. *How do work experiences differ with respect to age and sex three years after graduation?*
2. *What are the associations between work experiences*,* health*,* well-being*,* and intention to remain in the profession three years after graduating?*

## Methods

### Study design

This multicentre cross-sectional study included healthcare and social work professionals three years after graduating in biomedical science, dental hygiene, nursing, occupational therapy, physiotherapy, radiology nursing, and social work at six universities in Sweden. Three years after graduation was selected because participants were expected to have established themselves in professional practice, accumulated sufficient workplace experience, and transitioned beyond the initial adaptation period associated with entering the workforce [41]. The outcome concerned intention to remain in the profession, irrespective of employer or workplace. The study is part of a larger longitudinal research project [41] studying health-promoting factors during education and at two time points in early working life. The instruments (WEMS, SOC-13 and SHIS) have demonstrated satisfactory validity and reliability in previous studies [42–44].

### Sampling and participants

A total of 1,751 healthcare and social work professionals were invited to participate in a survey three years after graduation. The invitation was distributed via email, and 165 professionals completed the survey, yielding a response rate of 9.4%.

### Data collection

Data were collected between December 2024 and January 2025 using a web-based survey administered through esMaker NX3. The survey included items on general health and well-being, healthy lifestyle factors, health-promoting resources, and workplace experiences, as well as demographic variables such as age and sex. In total, the survey comprised 136 items and was available for approximately eight weeks. Three reminders were sent during the data collection period.

### Instruments

#### Work experience

*Work experience* was assessed using the work experience measurement scale (WEMS), a validated instrument measuring multiple dimensions of the psychosocial work environment [45]. The WEMS consists of six subscales: supportive work conditions (seven items), internal work experience (six items), autonomy (four items), time experience (three items), management (six items), and process of change (six items). Items are rated on a six-point Likert scale ranging from 1 (“disagree completely”) to 6 (“agree completely”), yielding a total score between 32 and 192. Higher scores indicate more positive workplace experiences. The WEMS has demonstrated satisfactory validity and reliability [42].

#### Health and well-being

*Health and well-being* were assessed through measures of SOC, perceived health, general health and well-being, and healthy lifestyle factors. SOC was measured using the sense of coherence 13-item scale (SOC-13) [21], which assesses comprehensibility (five items), manageability (four items), and meaningfulness (four items). Responses are given on a seven-point Likert scale, ranging from 1 (low agreement) to 7 (high agreement), with total scores between 13 and 91. Higher scores indicate a stronger sense of coherence. The SOC-13 has demonstrated strong psychometric properties [43].

*Perceived health* was measured using the 12-item salutogenic health indicator scale (SHIS), which captures cognitive, physical, and psychosomatic dimensions of health over the preceding four weeks [44]. Items are rated on a six-point Likert scale ranging from 1 (negative) to 6 (positive), resulting in total scores between 12 and 72. Higher scores reflect better perceived health. The SHIS has demonstrated strong psychometric properties, including high validity, and is considered a reliable instrument for evaluating salutogenic aspects of health [44].

*General health and perceived well-being* were assessed using two single-item questions (“In general, how would you describe your health?” and “In general, how would you describe your well-being?”). Responses were given on a five-point Likert scale ranging from poor (1) to excellent (5).

*Healthy lifestyle* factors were assessed using three items from the Swedish Public Health Survey [46] covering physical exercise (assessed on a six-point scale ranging from 0 min (1) to more than 2 h per week (6)), everyday physical activity (assessed on a seven-point scale ranging from 0 min (1) to more than 5 h per day (7)), and sleeping problems (assessed on a three-point scale ranging from no problems (1) to severe problems (3)).

#### Intention to remain in the profession

Intention to remain in the profession was measured using a single-item question rated on a four-point Likert scale, ranging from very unsure (1) to very sure (4).

### Data analysis

Descriptive statistics are presented as numbers and proportions or means and standard deviations for the total sample and stratified by sex. SHIS was analysed as a total score, while SOC-13 was analysed both as a total score and by its three subscales. Physical exercise and everyday physical activity were dichotomised based on duration, using cut-offs of 60 min and 150 min per week, respectively. Sleeping problems were dichotomised into no problems versus mild or severe problems. Mann–Whitney U tests, Fisher’s exact tests, or chi-square tests were used to analyse differences in characteristics between women and men, as appropriate. Non-parametric tests were applied as data were not normally distributed.

Mean WEMS scores, including its six subscales, were calculated for the total sample and stratified by sex and age group. Age was categorised into three groups: ≤ 29 years, 30–36 years, and ≥ 37 years. Group differences were analysed using Mann–Whitney U-tests and Kruskal–Wallis tests. General health and well-being were analysed across sex and the three age groups using mean values and standard deviations. Intention to remain in the profession was also analysed across sex and age. For these analyses, the variable was dichotomised into high intention (1 = “quite sure” or “very sure”) and low intention (0 = “quite unsure” or “very unsure”). Group differences were assessed using non-parametric tests. Regression analyses were performed to examine associations between the total WEMS score and general health, well-being, and intention to remain in the profession. Linear regression was applied for general health and well-being, whereas binary logistic regression was applied to intention to remain in the profession, dichotomised as high versus low intention. All models were adjusted for age and sex. The level of statistical significance was set at *p* < 0.05. All statistical analyses were performed using IBM SPSS Statistics for Windows, version 31.

## Results

In total, 165 participants (135 women, 28 men, and 2 who preferred not to specify) responded to the survey and were included in the overall analyses. Sex-stratified and sex-adjusted analyses were based on participants who reported sex (*n* = 163). Background characteristics including age, distribution across educational programmes, SOC, SHIS, and healthy lifestyle factors are presented in Table 1. The mean age was 35.0 years, with significantly higher age among men than women (36.6 years vs. 34.8, *p* = 0.045). The mean SHIS score was 46.7, with significantly higher scores among men than women (51.5 vs. 45.9, *p* = 0.048). Men also had higher total SOC scores than women, but the difference was statistically significant only for the comprehensibility subscale (25.0 vs. 22.4, *p* = 0.036). Almost half of the participants reported engaging in high-intensity physical activity, and more than half reported no sleep problems. No statistically significant differences in healthy lifestyle factors were observed between women and men.

**Table 1:** Characteristics of participants stratified by sex

	Total *n* = 165	Women *n* = 135	Men *n* = 28	*p*-value
Age, mean (SD)	35.0 (8.5)	34.8 (8.7)	36.6 (7.2)	**0.045** ^a^
Educational programme, n (%)				
Occupational therapist	12 (7.3)	9 (6.7)	2 (7.1)	*
Biomedical laboratory scientist	7 (4.2)	5 (3.7)	2 (7.1)	
Physiotherapist	11 (6.7)	8 (5.9)	3 (10.7)	
Diagnostic radiology nurse	3 (1.8)	2 (1.5)	1 (3.6)	
Registered nurse	97 (58.8)	85 (63.0)	12 (42.9)	
Social worker	34 (20.6)	25 (18.5)	8 (28.6)	
Registered dental hygienist	1 (0.6)	1 (0.7)	0 (0)	
SHIS total, mean (SD)	46.7 (12.8)	45.9 (13.1)	51.5 (8.5)	**0.048** ^**a**^
SOC-13 total, mean (SD)	62.9 (11.9)	62.6 (11.6)	64.9 (11.5)	0.443^a^
Comprehensibility	22.9 (5.6)	22.4 (5.6)	25.0 (4.7)	**0.036** ^**a**^
Meaningfulness	20.6 (4.1)	20.8 (3.9)	19.9 (4.7)	0.383^a^
Manageability	19.3 (4.3)	19.2 (4.2)	20.0 (4.0)	0.370^a^
Healthy lifestyle factors, n (%)				
Physical activity				
Physical exercise > 60 min/week	76 (46.3)	62 (46.3)	14 (50.0)	0.719^b^
Everyday physical activity > 150 min/week	94 (57.3)	75 (56.0)	19 (67.9)	0.246^b^
No sleep problems	85 (51.8)	68 (50.7)	16 (57.1)	0.538^b^

a: Mann–Whitney U

b: χ^2^-test

SHIS: The Salutogenic Health Indicator Scale; SOC: The Sense of Coherence scale

*: No significance test performed due to few participants in each group

Table 2 presents mean WEMS scores, including its six dimensions, for the total sample and stratified by sex and age. Men reported higher WEMS scores than women, but the difference was not statistically significant. No statistically significant differences in WEMS scores were observed across age groups. Table 2 also shows general health and well-being and intention to remain in the profession. Younger participants reported significantly higher scores on general health (*p* < 0.001) and well-being (*p* = 0.002) compared to the two older age groups. The intention to remain in the profession was significantly lower among participants aged 30–36 years compared with both younger and older participants (*p* = 0.042).

**Table 2:** Mean and standard deviation of the work experience measurement scale (WEMS) with six dimensions as well as general health, well-being, and intention to remain in the profession. Stratified by sex and age

		Sex			Age			
	Total *n* = 165	Women *n* = 135	Men *n* = 28	*p*-value	≤ 29 years *n* = 49	30–36 years *n* = 64	≥ 37 years *n* = 52	*p*-value
WEMS total, mean (SD)	136.1 (25.1)	135.3 (24.6)	140.4 (26.7)	0.544^a^	137.1 (23.2)	134.7 (26.8)	136.9 (25.3)	0.838^c^
Supportive work conditions	32.8 (6.3)	32.6 (6.4)	33.9 (5.7)	0.403^a^	33.4 (6.0)	32.4 (6.3)	32.7 (6.7)	0.620^c^
Internal work experiences	29.1 (4.6)	29.1 (4.5)	29.1 (4.9)	0.907^a^	29.2 (4.1)	28.4 (5.0)	29.9 (4.5)	0.325^c^
Autonomy	15.4 (4.1)	15.3 (3.9)	16.0 (4.6)	0.423^a^	15.3 (3.8)	15.2 (4.1)	15.9 (4.2)	0.801^c^
Time experience	11.4 (4.2)	11.3 (4.2)	11.9 (3.9)	0.495^a^	11.3 (4.2)	11.1 (4.3)	11.8 (4.2)	0.708^c^
Management	25.1 (7.5)	24.9 (7.5)	26.2 (7.7)	0.382^a^	25.2 (7.8)	24.8 (7.7)	25.3 (7.2)	0.967^c^
Process of change	22.7 (7.8)	22.5 (7.6)	23.8 (8.4)	0.388^a^	23.1 (6.9)	22.6 (8.6)	22.4 (7.5)	0.778^c^
General health, mean (SD)	3.3 (0.9)	3.3 (0.9)	3.3 (1.0)	0.724^a^	3.6 (0.7)	3.3 (0.9)	3.0 (1.0)	**< 0.001** ^**c**^
General well-being, mean (SD)	3.3 (1.0)	3.2 (1.0)	3.2 (0.9)	0.924^a^	3.7 (0.8)	3.2 (1.1)	3.0 (1.0)	**0.002** ^**c**^
Intention to remain in the profession, high, n (%)	127 (77.0)^*^	103 (76.3)	23 (82.1)	0.410^b^	39 (79.6)	43 (67.2)	45 (86.5)	**0.042** ^**b**^

a: Mann–Whitney U, b: χ^2^-test, c: Kruskal–Wallis, ^*^: One participant with high intention to remain in the profession had missing data on sex

As shown in Table 3, a higher total WEMS score was significantly associated with better general health (β = 0.009, *p* < 0.001), well-being (β = 0.012, *p* < 0.001), and intention to remain in the profession (OR = 1.036, *p* < 0.001) (Table 3). These associations remained significant after adjustments for age and sex; however, the univariate association between sex and the outcome variables was not statistically significant (Additional Tables 1 and 2).

**Table 3:** Multivariate associations between the work experience measurement scale (WEMS) and dependent variables general health, general well-being, and intention to remain in the profession

	Dependent variables								
	General health^a^			General well-being^a^			Intention to remain in the profession^b^		
	β (95% CI)	R^2^	p-value	β (95% CI)	R^2^	p	Exp(B) (95% CI)	R^2^*	p-value
WEMS total	0.009 (0.004–0.015)	0.072	**< 0.001**	0.012 (0.006–0.018)	0.096	**< 0.001**	1.036 (1.018–1.054)	0.173	**< 0.001**
Model 1	0.008 (0.003–0.014)	0.106	**0.002**	0.012 (0.006–0.018)	0.116	**< 0.001**	1.036 (1.018–1.054)	0.176	**< 0.001**
Supportive work conditions	0.032 (0.011–0.054)	0.053	**0.003**	0.045 (0.022–0.069)	0.082	**< 0.001**	1.153 (1.082–1.228)	0.195	**< 0.001**
Model 1	0.027 (0.006–0.048)	0.099	**0.014**	0.043 (0.019–0.067)	0.106	**< 0.001**	1.156 (1.082–1.235)	0.203	**< 0.001**
Internal work experiences	0.036 (0.006–0.065)	0.033	**0.019**	0.054 (0.021–0.086)	0.060	**0.002**	1.321 (1.185–1.472)	0.303	**< 0.001**
Model 1	0.033 (0.004–0.062)	0.091	**0.027**	0.053 (0.020–0.086)	0.093	**0.002**	1.332 (1.187–1.494)	0.313	**< 0.001**
Autonomy	0.044 (0.010–0.077)	0.038	**0.012**	0.045 (0.007–0.083)	0.032	**0.021**	1.076 (0.983–1.177)	0.023	0.113
Model 1	0.045 (0.012–0.078)	0.103	**0.008**	0.045 (0.007–0.083)	0.067	**0.020**	1.078 (0.983–1.183)	0.038	0.109
Time experience	0.047 (0.014–0.079)	0.047	**0.005**	0.057 (0.020–0.093)	0.056	**0.003**	1.118 (1.021–1.223)	0.056	**0.016**
Model 1	0.044 (0.011–0.076)	0.102	**0.009**	0.056 (0.020–0.093)	0.074	**0.003**	1.105 (1.008–1.211)	0.056	**0.033**
Management	0.016 (-0.003–0.034)	0.017	0.095	0.025 (0.005–0.045)	0.035	**0.017**	1.065 (1.014–1.119)	0.059	**0.012**
Model 1	0.013 (-0.005–0.031)	0.074	0.150	0.024 (0.004–0.044)	0.066	**0.020**	1.062 (1.011–1.116)	0.068	**0.017**
Process of change	0.011 (-0.007–0.029)	0.009	0.227	0.016 (-0.004–0.036)	0.015	0.117	1.068 (1.017–1.122)	0.067	**0.009**
Model 1	0.008 (-0.010–0.025)	0.054	0.396	0.016 (-0.004–0.036)	0.015	0.117	1.066 (1.014–1.120)	0.074	**0.012**

a: Linear regression, b: logistic regression, *: Nagelkerke R square. Model 1 adjusted for age and sex

Several WEMS dimensions were significantly associated with general health and well-being and the intention to remain in the profession (Table 3). Autonomy was significantly associated with general health and well-being, but not with the intention to remain in the profession. Moreover, management was not significantly associated with general health but with general well-being and the intention to remain in the profession. The highest effect sizes and coefficients of determination were found between intention to remain in the profession and supportive work conditions (OR = 1.153, *p* < 0.001) as well as internal work experiences (OR = 1.321, *p* < 0.001).

## Discussion

The present study showed that more positive work experiences were associated with better general health and well-being and with a stronger intention to remain in the profession among healthcare and social work professionals three years after graduation. Notably, supportive work conditions and internal work experiences demonstrated the strongest associations with the intention to remain in the profession, while autonomy was associated with general health and well-being but not with the intention to remain in the profession. The lack of association between perceived autonomy and the intention to remain in the profession suggests that, during early working life, autonomy may be more closely related to health promotion than to professionals’ intention to remain in the profession. Management, in turn, was associated with well-being and the intention to remain in the profession, but not with general health. Together, these findings suggest that different aspects of the work environment may influence health, well-being, and professionals’ intention to remain in the profession in distinct ways early in working life.

The strong associations between total WEMS scores and all three outcomes highlight the importance of psychosocial work experiences early in professional life. As WEMS captures both structural and relational aspects of the work environment, the findings suggest that these factors jointly shape how healthcare and social work professionals perceive their health, well-being, and long-term sustainability in the profession. Supportive work conditions and positive internal work experiences appear central for the intention to remain in the profession, in line with previous research [16, 47]. Supportive work conditions and positive internal work experiences, thus, play an important role for the intention to remain in the profession. These dimensions reflect access to social support, opportunities for participation in decision-making, and sense of meaningfulness and competence - factors consistently linked to job satisfaction and long-term commitment in previous research [34, 48]. Strengthening these aspects of the work environment may therefore be especially important for promoting the intention to remain in the profession among early-career professionals.

The differentiated associations observed across WEMS dimensions provide further insight into how the work environment relates to specific outcomes. The findings suggest that autonomy may primarily support internal psychological functioning rather than decisions to stay in the profession. Interpreted, in light of self-determination theory [49, 50], autonomy contributes to the fulfilment of basic psychological needs, which in turn enhances well-being and resilience. In contrast, management was associated with well-being and intention to remain in the profession, but not with health, indicating that leadership and organisational practices may play a more prominent role in shaping healthcare and social work professionals’ perceptions of their work environment and their intentions to remain in the profession. This distinction suggests that early-career professionals may differentiate between internal experiences of motivation and external organisational conditions when evaluating their future in the profession [47, 48].

Participants aged 30-36 years reported a significantly lower intention to remain in the profession than both younger and older groups, suggesting that this age range may represent a particularly vulnerable period during which targeted strategies to support the intention to remain in the profession may especially effective. This may reflect increasing exposure to work-related stressors, unmet expectations, or challenges in balancing professional and personal demands at this stage of career development [16, 47]. Identifying this group at risk of leaving the profession underscores the importance of organisational initiatives that strengthen supportive leadership and collegial support, thereby promoting well-being and reducing psychological distress among professionals [34].

No significant differences between men and women were observed in healthy lifestyle factors, suggesting that health-related behaviours may be relatively similar among men and women in this population. This finding points to the relevance of workplace interventions that address common rather than gender-specific health behaviours, particularly in professions characterised by shared working conditions and demands.

The findings have several practical implications for promoting sustainable working lives in healthcare and social work. First, the strong relationship between work experiences and well-being highlights the value of systematically assessing and improving working conditions [42]. Second, the importance of supportive work conditions underscores the key role of first-line managers in fostering environments characterised by trust, participation, and support that may strengthen professionals’ intention to remain in the profession [51]. Third, the variation in how different WEMS dimensions relate to outcomes suggests that tailored, rather than generic, interventions are needed [52]. Finally, the lower intention to remain in the profession among participants highlights the need for timely organisational strategies that support continued engagement and career sustainability.

Overall, the findings emphasise that work experiences are closely linked to both well-being and the intention to remain in the profession. Creating work environments that support positive work experiences, psychological resources, and meaningful engagement is therefore essential for sustaining a healthy and stable workforce in healthcare and social work. The present findings align with recommendations from WHO, ILO and EU-OSHA, which emphasise supportive psychosocial working environments, leadership and employee participation as important prerequisites for occupational health, well-being and workforce sustainability.

### Strengths and limitations

This study has several strengths. The multicentre design, including participants from six universities across Sweden, enhances the diversity of the sample and supports the generalisability of the findings within a Swedish context. The use of well-established and validated instruments, such as WEMS [42, 45], SOC-13 [43], and SHIS [44], strengthens the reliability and validity of the measurements. In addition, the survey covered a broad range of domains, including work experience, health, well-being, and healthy lifestyle factors, enabling a comprehensive assessment of participants’ working life and health. The inclusion of general health and well-being items derived from the Swedish National Public Health Survey [46] further supports the relevance and comparability of these measures. Moreover, the study’s integration within a larger longitudinal project is also a strength, as it provides opportunities to examine changes over time in future analyses.

The study also has limitations. The low response rate (9.4%) raises concerns about potential response bias and limits the representativeness of the sample, which may affect the generalisability of the findings. It is possible that participants differed systematically from non-responders, for example in terms of health status or work experiences. Furthermore, the cross-sectional design prevents conclusions about causal relationships between work experiences and health-related outcomes. All data were self-reported, which may introduce biases such as social desirability bias and recall bias, potentially affecting the accuracy of the results. In addition, the relatively small number of men in the sample should be considered, and findings related to sex differences should therefore be interpreted with caution.

## Conclusions

This study showed that work experiences in the early-career phase were associated with general health and well-being and with the intention to remain in the profession among healthcare and social work professionals. The lower intention to remain in the profession among professionals aged 30-36 years highlights a potentially critical period in need of targeted organisational support. Supportive work conditions and positive internal work experiences were particularly important for the professionals’ intention to remain in the profession, while management was primarily associated with both well-being and the intention to remain in the profession. These findings suggest that different aspects of the work environment contribute in distinct ways to health, well-being, and professional sustainability in early working life. From a professional, health, and social policy perspective, the results point to the importance of integrating early-career support, sustainable leadership, and supportive work conditions into workforce strategies across healthcare professions and social work. Such policy and organisational strategies may support professionals’ well-being and intention to remain in their profession, while also contributing to a more sustainable future workforce within health and social care sectors.

## Supplementary Information

Below is the link to the electronic supplementary material.

Supplementary Material 1

## Data Availability

The datasets generated and analysed during the current study are not publicly available but are available from the corresponding author upon reasonable request.
