# Supplementary material for: Associations between work experiences, health, well-being, and intention to remain in the profession among healthcare and social work professionals – a multicentre cross-sectional study
Source: BMC Health Serv Res. 2026 Sep 17;26:1284. doi: 10.1186/s12913-026-15575-y (PMC13589342; doi:10.1186/s12913-026-15575-y)
Supplement: Supplementary file 1 [file 12913_2026_15575_MOESM1_ESM.docx]

Table S1. Multivariate associations between the work experience measurement scale (WEMS) and general health.

|  | | | General health^a^ | | | | | | | |
| --- | --- | --- | --- | --- | --- | --- | --- | --- | --- | --- |
|  | | | Crude | |  | Model 1 | |  | Model 2 | |
|  | | | β (95% CI) | p-value |  | β (95% CI) | p-value |  | β (95% CI) | p-value |
| WEMS total, n = 159 | | | 0.009 (0.004–0.015) | **<0.001** |  | 0.009 (0.004-0.014) | **<0.001** |  | 0.008 (0.003–0.014) | **0.002** |
|  | Age | |  |  |  | −0.020 (−0.036– (−0.005)) | **0.008** |  | −0.021 (−0.037– (−0.006)) | **0.008** |
|  | Sex (Men) | |  |  |  |  |  |  | 0.014 (−0.339–0.367) | 0.938 |
| Supportive work conditions, n = 163 | | | 0.032 (0.011–0.054) | **0.003** |  | 0.030 (0.009–0.051) | **0.005** |  | 0.027 (0.006–0.048) | **0.014** |
|  | Age | |  |  |  | −0.023 (−0.038– (0.007)) | **0.004** |  | −0.024 (−0.040– (−0.008)) | **0.003** |
|  | Sex (Men) | |  |  |  |  |  |  | 0.057 (−0.295–0.410) | 0.749 |
| Internal work experiences, n = 164 | | | 0.036 (0.006–0.065) | **0.019** |  | 0.036 (0.007–0.065) | **0.016** |  | 0.033 (0.004–0.062) | **0.027** |
|  | Age | |  |  |  | −0.025 (−0.040– (−0.009) | **0.002** |  | −0.026 (−0.042– (−0.010) | **0.001** |
|  | Sex (Men) | |  |  |  |  |  |  | 0.084 (−0.271–0.439) | 0.642 |
| Autonomy, n = 164 | | | 0.044 (0.010–0.077) | **0.012** |  | 0.044 (0.011–0.077) | **0.009** |  | 0.045 (0.012–0.078) | **0.008** |
|  | | Age |  |  |  | −0.025 (−0.041– (−0.009)) | **0.002** |  | −0.027 (−0.042– (−0.011)) | **0.001** |
|  | | Sex (Men) |  |  |  |  |  |  | 0.052 (−0.302–0.406) | 0.773 |
| Time experience, n = 163 | | | 0.047 (0.014–0.079) | **0.005** |  | 0.048 (0.016–0.080) | **0.003** |  | 0.044 (0.011–0.076) | **0.009** |
|  | Age | |  |  |  | −0.025 (−0.041– (−0.010)) | **0.002** |  | −0.027 (−0.042– (−0.011)) | **0.001** |
|  | Sex (Men) | |  |  |  |  |  |  | 0.060 (−0.295–0.415) | 0.332 |
| Management, n = 163 | | | 0.016 (−0.003–0.034) | 0.095 |  | 0.015 (−0.003–0.033) | 0.100 |  | 0.013 (−0.005–0.031) | 0.150 |
|  | Age | |  |  |  | −0.024 (−0.040– (−0.008)) | **0.003** |  | −0.026 (−0.042– (−0.010)) | **0.002** |
|  | Sex (Men) | |  |  |  |  |  |  | 0.068 (−0.291–0.427) | 0.709 |
| Process of change, n = 161 | | | 0.011 (−0.007–0.029) | 0.227 |  | 0.010 (−0.008–0.027) | 0.271 |  | 0.008 (−0.010–0.025) | 0.396 |
|  | Age | |  |  |  | −0.021 (−0.037– (−0.005)) | **0.009** |  | −0.023 (−0.039– (−0.007)) | **0.006** |
|  | Sex (Men) | |  |  |  |  |  |  | 0.036 (−0.328–0.401) | 0.844 |

a: Linear regression

Table S2. Multivariate associations between the work experience measurement scale (WEMS) and general well-being.

|  | | General well-being^a^ | | | | | | | |
| --- | --- | --- | --- | --- | --- | --- | --- | --- | --- |
|  | | Crude | |  | Model 1 | |  | Model 2 | |
|  | | β (95% CI) | p-value |  | β (95% CI) | p-value |  | β (95% CI) | p-value |
| WEMS total, n = 159 | | 0.012 (0.006–0.018) | **<0.001** |  | 0.012 (0.006–0.018) | **<0.001** |  | 0.012 (0.006–0.018) | **<0.001** |
|  | Age |  |  |  | −0.018 (−0.036–0.000) | **0.047** |  | −0.017 (−0.035–0.000) | 0.053 |
|  | Sex (Men) |  |  |  |  |  |  | −0.040 (−0.435–0.355) | 0.843 |
| Supportive work conditions, n = 163 | | 0.045 (0.022–0.069) | **<0.001** |  | 0.043 (0.019–0.067) | **<0.001** |  | 0.043 (0.019–0.067) | **<0.001** |
|  | Age |  |  |  | −0.019 (−0.036– (−0.001)) | **0.034** |  | −0.019 (−0.036– (-0.001)) | **0.038** |
|  | Sex (Men) |  |  |  |  |  |  | −0.008 (−0.402–0.385) | 0.966 |
| Internal work experiences, n = 164 | | 0.054 (0.021–0.086) | **0.002** |  | 0.053 (0.021–0.086) | **0.001** |  | 0.053 (0.020–0.086) | **0.002** |
|  | Age |  |  |  | −0.022 (−0.039– (−0.004)) | **0.015** |  | −0.022 (−0.039– (−0.004)) | **0.016** |
|  | Sex (Men) |  |  |  |  |  |  | 0.041 (−0.356–0.438) | 0.839 |
| Autonomy, n = 164 | | 0.045 (0.007–0.083) | **0.021** |  | 0.046 (0.008–0.083) | **0.017** |  | 0.045 (0.007–0.083) | **0.020** |
|  | Age |  |  |  | −0.022 (−0.040– (−0.005)) | **0.014** |  | −0.022 (−0.040– (−0.004)) | **0.015** |
|  | Sex (Men) |  |  |  |  |  |  | 0.010 (−0.394–0.414) | 0.962 |
| Time experience, n = 163 | | 0.057 (0.020–0.093) | **0.003** |  | 0.057 (0.021–0.093) | **0.002** |  | 0.056 (0.020–0.093) | **0.003** |
|  | Age |  |  |  | −0.023 (−0.041– (−0.006)) | **0.010** |  | −0.023 (−0.041– (-0.005)) | **0.011** |
|  | Sex (Men) |  |  |  |  |  |  | 0.019 (−0.380–0.419) | 0.923 |
| Management, n = 163 | | 0.025 (0.005–0.045) | **0.017** |  | 0.024 (0.004–0.044) | **0.019** |  | 0.024 (0.004–0.044) | **0.020** |
|  | Age |  |  |  | −0.021 (−0.039– (−0.003)) | **0.020** |  | −0.021 (−0.039– (−0.003)) | **0.022** |
|  | Sex (Men) |  |  |  |  |  |  | 0.011 (−0.392–0.415) | 0.955 |
| Process of change, n = 161 | | 0.016 (−0.004–0.036) | 0.117 |  | 0.015 (−0.005–0.035) | 0.143 |  | 0.014 (−0.006–0.034) | 0.164 |
|  | Age |  |  |  | -0.018 (−0 036–0.000) | **0.050** |  | −0.018 (−0 036–0.000) | 0.055 |
|  | Sex (Men) |  |  |  |  |  |  | −0.019 (−0.432–0.395) | 0.929 |

a: Linear regression.
